# Management of children with sepsis and septic shock: a survey among pediatric intensivists of the Réseau Mère-Enfant de la Francophonie

**DOI:** 10.1186/2110-5820-3-7

**Published:** 2013-03-14

**Authors:** Miriam Santschi, Francis Leclerc

**Affiliations:** 1 Département de pédiatrie, Université de Sherbrooke, Centre Hospitalier Universitaire de Sherbrooke, 3001, 12 avenue Nord, Sherbrooke, Qc, J1H 5N4, Canada; 2 Service de réanimation pédiatrique, Centre Hospitalier Régional Universitaire de Lille France - Univ Lille Nord de France, UDSL, 2694, Lille, EA, F-59000, France

**Keywords:** Sepsis, Septic shock, Child, Pediatric intensive care unit

## Abstract

**Background:**

Pediatric sepsis represents an important cause of mortality in pediatric intensive care units (PICU). Although adherence to published guidelines for the management of severe sepsis patients is known to lower mortality, actual adherence to these recommendations is low. The aim of this study was to describe the initial management of pediatric patients with severe sepsis, as well as to describe the main barriers to the adherence to current guidelines on management of these patients.

**Methods:**

A survey using a case scenario to assess the management of a child with severe sepsis was designed and sent out to all PICU medical directors of the 20 institutions member of the “Réseau Mère- Enfant de la Francophonie”. Participants were asked to describe in detail the usual management of these patients in their institution with regard to investigations, fluid and catecholamine management, intubation, and specific treatments. Participants were also asked to identify the main barriers to the application of the Surviving Sepsis Campaign guidelines in their center.

**Results:**

Twelve PICU medical directors answered the survey. Only two elements of the severe sepsis bundles had a low stated compliance rate: “maintain adequate central venous pressure” and “glycemic control” had a stated compliance of 8% and 25% respectively. All other elements of the bundles had a reported compliance of over 90%. Furthermore, the most important barriers to the adherence to Surviving Sepsis Campaign guidelines were the unavailability of continuous central venous oxygen saturation (ScvO_2_) monitoring and the absence of a locally written protocol.

**Conclusions:**

In this survey, pediatric intensivists reported high adherence to the current recommendations in the management of pediatric severe sepsis regarding antibiotic administration, rapid fluid resuscitation, and administration of catecholamines and steroids, if needed. Technical difficulties in obtaining continuous ScvO_2_ monitoring and absence of a locally written protocol were the main barriers to the uniform application of current guidelines. We believe that the development of locally written protocols and of specialized teams could add to the achievement of the goal that every child in sepsis should be treated according to the latest evidence to heighten his chances of survival.

## Background

Pediatric sepsis and septic shock represent an important cause of mortality in pediatric intensive care units (PICU) and one of the leading causes of childhood mortality worldwide. In developed countries, mortality from septic shock ranges between 10% and 50% among children [1-3]. Guidelines and recommendations for the management of pediatric septic shock patients have been published since 2002 by the American College of Critical Care Medicine – Pediatric Advanced Life Support [4] and reinforced by a pediatric section in the first Surviving Sepsis Campaign guidelines in 2004 [5], as well as regular updates thereafter [1,6]. The objectives of these recommendations were to standardize patient care and further reduce mortality and morbidity in pediatric sepsis. These guidelines represent best clinical practice; however, stronger evidence is lacking to confirm the components of these recommendations; almost all levels of references and recommendations in pediatric septic shock treatment are low [6-8].

Adherence to these recommendations is known to lower mortality in pediatric septic patients as shown in a study by Han et al. (mortality 8% vs. 38%) [9]. However, observational studies have shown low adherence to these recommendations: only 8-30% of pediatric patients presenting with septic shock or severe sepsis will be managed according to the guidelines [2,9,10]. The literature identifies several barriers that limit adherence to current guidelines, including lack of early recognition of severe sepsis and septic shock as well as treatment delay, difficulties in obtaining adequate vascular access and advanced airway management, central venous pressure and central venous oxygen saturation (ScvO_2_) monitoring, shortage of health care providers, absence of goals and treatment protocols, difficulties in obtaining specialized transport and access to pediatric intensive care beds, as well as educational gaps [2,7-9,11,12].

The purpose of this study was to describe initial management of pediatric severe sepsis and septic shock patients as stated by a diverse group of pediatric intensivists, as well as to describe the main barriers to the adherence to current guidelines on the management of pediatric patients with severe sepsis or septic shock.

## Methods

### Study design

A survey on the management of pediatric patients with severe sepsis and septic shock was designed. Participants were asked to describe the typical management of these patients in their intensive care unit. Questions addressing investigations, fluid and catecholamine management, intubation, and specific treatments (antibiotics, steroids, transfusions, and insulin) were answered. Participants also were asked to identify the main barriers to the application of the Surviving Sepsis Campaign guidelines in their center [6].

### Development of written questionnaire

The questionnaire was developed by two investigators (MS and FL). A case scenario was developed and participants were asked to state the usual management of that virtual patient in their intensive care unit (ICU). The case scenario was a 16-month-old patient, with no previous medical history, presenting with a 2-day history of upper respiratory tract infection and fever. The patient’s condition had worsened in the last hour, and he was in septic shock on arrival to the emergency room: he was tachycardic at 185 beats/min, had a blood pressure of 67/35 mmHg, and was febrile at 39.8°C. He was lethargic and had prolonged capillary refill time and mottled skin, with a dozen petechiae on both legs. Participants were asked to provide details about the investigations that they would undertake for this patient, their typical fluid and catecholamine management, intubation timing and medications used, as well as steroid, transfusion, and insulin indications (the questionnaire is available on request).

### Study population

The survey was sent out to all PICU medical directors of the 20 institutions member of the “Réseau Mère Enfant de la Francophonie” spread over four continents. We decided *a priori* to exclude physicians working exclusively in a neonatal or an adult intensive care unit. Furthermore, only medical directors were asked to participate. The survey was sent out in November 2010 by e-mail. Data collection was closed in March 2011. The questionnaire was developed and administered in French.

### Statistical analysis

Categorical data are expressed as frequencies (%), whereas continuous data are expressed as medians (range) as distribution was non-normal. Compliance to the two bundles of the Surviving Sepsis Campaign guidelines was calculated.

## Results

Thirteen physicians answered the questionnaire; however, one was excluded because he was medical director of a neonatal intensive care unit. Therefore, the results of 12 PICU medical directors, representing 60% of the member institutions of the “Réseau Mère-Enfant de la Francophonie,” are reported in this study. They represent PICUs from France (n = 6), Canada (n = 3), Lebanon (n = 1), Madagascar (n = 1), and Switzerland (n = 1). The median number of beds in the PICUs is 15 (range: 6–24 beds). All but one are combined medical and surgical PICUs. General characteristics of participating PICUs are presented in Table 1.

**Table 1:** Participating pediatric intensive care unit characteristics (n = 12)

	
No. of beds in PICU^a^	15 beds (6–24)
PICU population	
Pediatric medical	12 (100)
Pediatric cardiac surgery	5 (42)
Pediatric neurosurgery	6 (50)
Pediatric other kind of surgery	10 (83)
Pediatric burn unit	5 (42)
Pediatric trauma unit	7 (58)
Combined PICU and NICU	5 (42)
Combined PICU and adult ICU	0
% neonates per unit^a^	1% (0–45)
No. of admissions per year	
1–300 admissions/year	1 (8)
301–600 admissions/year	5 (42)
601–900 admissions/year	5 (42)
901–1200 admissions/year	1 (8)

Data are number (percentage) unless otherwise indicated.

^a^Median (range).

*PICU* pediatric intensive care unit; *NICU* neonatal intensive care unit; *ICU* intensive care unit.

**Figure 1:**
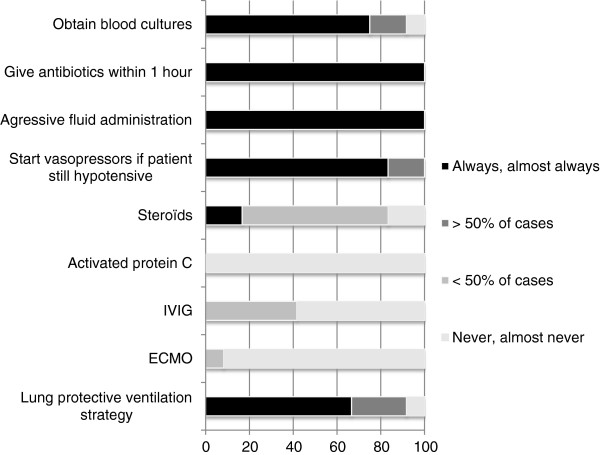
Interventions used in management of pediatric septic shock (in percentage).

Figure 1 reports how frequently different interventions were reported as being used in these PICUs while treating a pediatric patient in severe sepsis or septic shock. Regarding adherence to the severe sepsis bundles [13], only one PICU reported compliance to all the components of the first severe sepsis bundle (sepsis resuscitation bundle) and three PICUs reported compliance to all elements of the second severe sepsis bundle (sepsis management bundle). One element in each bundle had relatively low compliance, explaining the low total compliance to the bundles. In the sepsis resuscitation bundle, for the element “maintain adequate central venous pressure (CVP),” only one center reported aiming for a CVP over 8 mmHg. For the sepsis management bundle, only three centers reported using insulin in case of hyperglycemia (glycemia 10.5 mmol/L). All the other elements of the two bundles had stated compliance rates >90%.

Investigations used to determine the severity of sepsis are given in Table 2. When asked if different results to some of those investigations would have influenced the patient’s management, 10 centers (83%) reported that a lactate of 5 mmol/L would have influenced their management and 11 centers (92%) reported that a ScvO_2_ of 55% would have changed the patient’s care.

**Table 2:** Investigations to determine sepsis severity and parameters used to follow clinical response to fluid resuscitation

	
Investigations used to determine sepsis severity n(%)	
Serum lactate	11 (92%)
Arterial or venous blood gas	11 (92%)
Central venous saturation (ScVO_2_)	9 (75%)
Echocardiography	8 (67%)
Chest X-Ray	7 (58%)
Complete Blood count	7 (58%)
C reactive protein	4 (33%)
Coagulation tests	3 (25%)
Sedimentation rate	1 (8%)
Other	2 (17%)
Parameters used to follow response to fluid resuscitation n(%)	
Physical exam and vital signs	11 (92%)
Urine output	11 (92%)
Central Venous Pressure Monitoring	6 (50%)
Echocardiography	5 (42%)
Central Venous Saturation (ScvO2)	2 (17%)
Continuous monitoring of CO	1 (8%)
NIRS	1 (8%)
Swan Ganz	0
Other	2 (8%)

*CO* cardiac output; *NIRS* near-infrared spectroscopy.

All centers reported using crystalloids (normal saline or Ringer lactate) to initiate fluid resuscitation in septic pediatric patients. None reported using colloids as a first choice. Parameters used to monitor clinical response to fluid resuscitation are reported in Table 2. More than half of the centers (58%) reported considering catecholamines if the patient’s condition has not improved after 40–60 mL/kg of fluid resuscitation; 25% would consider it after 20–40 mL/kg and 8% after 60–80 mL/kg; finally, 8% consider catecholamines based on echocardiography results. When considering adding catecholamines, five centers (42%) reported they would start norepinephrine, three centers (25%) dopamine, two centers (17%) dobutamine, and one center (8%) epinephrine.

Intubation would be considered in 83% of centers if the patient remained hemodynamically unstable or with an altered mental status persisting after fluid resuscitation. Two centers (17%) would intubate the patient on arrival or with the first fluid bolus. The medications chosen to intubate included atropine in 36% of centers and short-acting neuromuscular blocking agents in 80% of centers. Finally, concerning inducing agents, ketamine would be used in 64% of centers, 57% of those centers would use ketamine alone, 29% would use it in combination with opiates, and 14% in combination with etomidate. Worth noting, etomidate was used in 27% of centers to intubate a pediatric patient in septic shock (alone or in combination with ketamine or opiates). Only one center reported using benzodiazepines and none used propofol or barbiturates.

The vast majority of centers (92%) would administer low-dose steroids if the patient was in refractory shock after adequate fluid resuscitation and catecholamines. One center (8%) would give steroids on arrival. No center reported waiting on abnormal cortisol test results or performing an ACTH stimulation test before administering steroids.

In the event that the patient develops hyperglycemia (10.5 mmol/L blood glucose), 75% of centers would not start an insulin perfusion, 17% would start insulin without any protocol, and 8% would start insulin following a written protocol.

Participating centers also were asked to state their usual transfusion practice in septic shock patients once the patient’s condition was stabilized. The patient was on two catecholamines, had recovered normal perfusion, and had a urine output >1 mL/kg. His laboratory results showed a blood lactate level of 3 mmol/L and ScvO_2_ of 60%, and a hemoglobin level of 8.5 g/L. Three centers (27%) would transfuse this patient in his current state. Five centers (46%) reported their usual practice would be to try to optimize ScvO_2_ and would consider transfusing the patient if other strategies to improve ScvO_2_ fail. One center (9%) would try to improve the patient’s ScvO_2_ but would not transfuse him even if the strategies failed. Finally, two centers (18%) considered that this patient did not need any further treatment.

**Figure 2:**
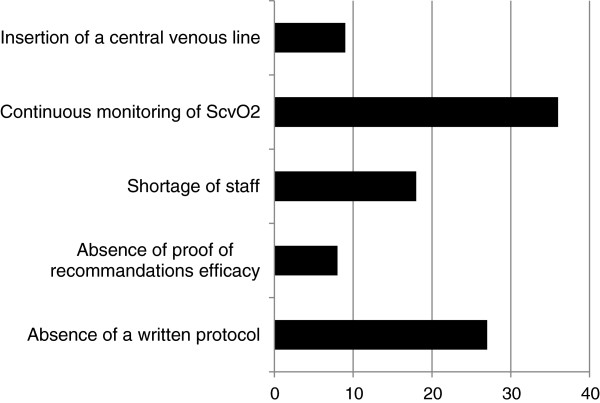
Barriers to the application of surviving sepsis campaign guidelines (in percentage).

The main barriers to the application of the Surviving Sepsis Campaign guidelines reported by participating PICUs are presented in Figure 2.

## Discussion

Overall, pediatric intensivists report a high level of adherence to most recommendations in the management of pediatric patients with severe sepsis and septic shock. The survey results show that administration of antibiotics in the first hour, aggressive fluid resuscitation, adding catecholamines, if needed, and administration of steroids in refractory shock were all used in the management of a child in sepsis suggesting that little or no controversy remains with respect to these intervention types. However, a lack of consensus appears across the 12 participating institutions regarding the type of vasopressor and intubation medications used, as well as the transfusion threshold.

Recommendations uniformly suggest initiating aggressive fluid resuscitation using either crystalloids or colloids with the addition of catecholamines in cases of fluid refractory shock [1,6]. The Surviving Sepsis Campaign guidelines suggest using dopamine as the first choice of support for pediatric patients with hypotension refractory to fluid resuscitation (recommendation Grade 2C) and dobutamine in patients with low cardiac output and elevated systemic vascular resistance states (recommendation Grade 2C) [6]. Norepinephrine and epinephrine are suggested for patients in dopamine-refractory shock depending on their vascular physiology at that time [6]. However, our data suggest a trend toward the use of norepinephrine as a first line vasopressor in pediatric septic shock patients in our study; this also was reported by Matt et al. [14] and Lampin et al. [15] The explanation of this trend is not clear but is likely due in part to a higher number of adverse events with dopamine than with norepinephrine [16].

A second point of interest revealed by this survey is that close to a third of centers reported using etomidate as an induction agent for intubation in septic shock patients. Popular as an induction agent, because it maintains cardiovascular stability, etomidate has been independently associated with increased mortality in patients with septic shock, possibly secondary to induced adrenal insufficiency [1]. Therefore, etomidate is currently not recommended as an adequate induction agent in patients with septic shock [1,17-19]. The favored inducing agent for intubation in pediatric patients with septic shock is ketamine, which was used in the vast majority (64%) of centers participating in this study.

Finally, opinions also differed regarding transfusion practices in a stabilized patient with low ScvO_2_ and low hemoglobin level. A certain number of centers reported aiming for a normal ScvO_2_ level in these patients with transfusions considered as an adequate tool to attain this goal. This strategy was consistent with the published data on advantages of early goal-directed therapy and reduction in mortality when aiming for a ScvO_2_ > 70% [20]. A pediatric study aiming for a ScvO_2_ > 70% that includes a strategy to transfuse patients to obtain this goal has shown an important reduction in mortality when this strategy was used (28-day mortality: 11.8% vs. 39.2%) [21]. On the other hand, some centers seem to avoid transfusions in stabilized patients even when ScvO_2_ remains suboptimal consistent with the results of TRIPICU study that showed that aiming for a lower hemoglobin level in stable pediatric intensive care patients did not worsen their outcome, even in patients with sepsis [22,23].

Nearly all centers reported using steroids in children with vasopressor refractory shock and suspected adrenal insufficiency as suggested by the Surviving Sepsis Campaign guidelines [6]. The recommendation against the use of Activated Protein C (Grade 1B) [6] also is uniformly accepted as none of the centers participating in this study reported using Activated Protein C. Furthermore, Extracorporeal Membrane Oxygenation (ECMO) was considered in a minority of centers as a last resort treatment in a patient that cannot be supported by conventional therapies, once again in accordance to current recommendations. Consensus regarding glycemic control was less obvious, reflecting the absence of published pediatric studies on the positive benefits of tight glycemic control in pediatric patients and the abundance of recommendations stating that long periods of hyperglycemia should be avoided, but not providing an optimal goal glucose level [6].

Overall compliance to the severe sepsis bundles were quite comparable to published adult data. In our study, 8% of PICU medical directors reported adherence to the first sepsis bundle compared with 10.9% in one adult study [24]. Reported compliance to the second bundle was 25% compared with 18.4% [24]. However, in our study, two single elements were responsible for the overall low compliance rate: goal central venous pressure to achieve and glycemic control. The compliance to all other elements was very high, >90%, reflecting a high overall adherence to actual Surviving Sepsis Campaign guidelines.

The main barriers reported by pediatric intensivists to the adherence to recommendations in the management of septic pediatric patients are consistent with other published data [2,9,11,12]. As expected, the availability of continuous ScvO_2_ monitoring and the absence of a locally written protocol for the management of septic shock patients were the most significant barriers identified. The challenge of an adequate vascular access not being reported as frequently as previously published studies [2,9] probably reflects the higher comfort with that technique among pediatric intensivists compared with emergency department physicians. Of note, one center reported the absence of proof of the Surviving Sepsis Campaign recommendations efficacy. This is an important subject of debate in the adult literature [25,26].

This study describes stated practice of management of pediatric patients with sepsis and septic shock. However, the study design records the perception that clinicians have of the way they would treat a “virtual” patient, which does not necessarily capture the true daily clinical practice. This is a limitation of surveys on stated practice patterns as reflected in a recent German study wherein it was shown that most septic adult patients did not receive recommended therapies, whereas a majority of ICU directors responsible for these patients reported that they adhered to these recommendations [27]. Furthermore, only one physician per center answered the questionnaire while management could potentially differ among physicians even in the same center. Finally, we asked pediatric intensivists to state their current management practices on a certain number of treatments that would be undertaken before the pediatric intensive care consultation (e.g., antibiotics and fluid resuscitation, for example) usually as the patient is still under the care of the emergency room physician.

## Conclusions

In this survey, pediatric intensivists reported quasi uniform adherence to current recommendations in the management of pediatric septic shock and severe sepsis with respect to antibiotic administration, rapid fluid resuscitation, and adding catecholamines and steroids, if needed. Overall, compliance to the Surviving Sepsis Campaign guidelines was only 8% for the resuscitation bundle and 25% for the sepsis management bundle, but this is largely due to a single recommendation in each bundle with very low compliance. Pediatric intensivists seemed divided on: the type of catecholamine to use as a first-line agent, the optimal medication for induction for intubation, and the adequate transfusion threshold to be used for these patients. Technical difficulties in obtaining continuous ScvO_2_ monitoring and absence of a locally written protocol were the main barriers reported to hinder the uniform application of all current guidelines. We believe that development of locally written protocols and of specialized teams could add to the achievement of the goal that every child in sepsis should be treated according to the latest evidence to heighten his chances of survival.

## Abbreviations

CO: Cardiac output; CVP: Central venous pressure; ECMO: Extracorporeal membrane oxygenation; ICU: Intensive care unit; IVIG: Intravenous immunoglobulins; NIRS: Near-infrared spectroscopy; PICU: Pediatric intensive care unit; ScvO2: Central venous oxygen saturation

## Competing interests

The authors declare that they have no competing interests.

## Authors’ contributions

Both authors contributed to the conception and design of the survey. MS analyzed the data and drafted the first version of the manuscript. FL critically revised the manuscript. All authors read and approved the final manuscript.
